# Bone Mass and Mineral Metabolism Alterations in Adult Celiac Disease: Pathophysiology and Clinical Approach

**DOI:** 10.3390/nu5114786

**Published:** 2013-11-22

**Authors:** Michele Di Stefano, Caterina Mengoli, Manuela Bergonzi, Gino Roberto Corazza

**Affiliations:** 1^st^ Department of Internal Medicine, University of Pavia, IRCCS “S.Matteo” Hospital Foundation, Pavia 27100, Italy; E-Mails: m.distefano@unipv.it (M.S.); caterina.mengoli@libero.it (C.M.); manuela.bergonzi@libero.it (M.B.)

**Keywords:** celiac disease, osteoporosis, gluten-free diet, bone densitometry

## Abstract

Osteoporosis affects many patients with celiac disease (CD), representing the consequence of calcium malabsorption and persistent activation of mucosal inflammation. A slight increase of fracture risk is evident in this condition, particularly in those with overt malabsorption and in postmenopausal state. The adoption of a correct gluten-free diet (GFD) improves bone derangement, but is not able to normalize bone mass in all the patients. Biomarkers effective in the prediction of bone response to gluten-free diet are not yet available and the indications of guidelines are still imperfect and debated. In this review, the pathophysiology of bone loss is correlated to clinical aspects, defining an alternative proposal of management for this condition.

## 1. Introduction

Osteoporosis is a condition characterized by low bone mass and micro-architectural deterioration of bone tissue resulting in enhanced bone fragility and an increase in fracture risk [1]. It affects more than 75 million people in developed countries, causing 8.9 million fractures annually worldwide. Osteoporotic fractures account for 2.8 million disability-adjusted life years annually: to make this even clearer, this index is higher than other conditions, such as breast cancer, gastric cancer and hypertension [2]. There is a general agreement in the literature that more than 75% of untreated adult celiac disease (CD) patients with an overt malabsorption syndrome at diagnosis suffer from a loss of bone mass [3,4,5,6,7,8,9], and this complication also affects about half the patients with subclinical CD, presenting with minimal, transient and apparently unrelated symptoms [3,9], or asymptomatic patients diagnosed because of their first-degree kinship [4].

Moreover, an increased prevalence of celiac disease in osteoporotic patients was reported [10,11,12] and, even if this result was not confirmed by others [13,14,15], all previous results show the importance of the problem from a clinical point of view. On the contrary, the value of active screening for CD in patients with otherwise unexplained bone loss is still under debate.

Accordingly, there is no doubt that CD is a condition at high risk for secondary osteoporosis, and the evaluation of bone mass and mineral metabolism is thus very important in the clinical management of these patients. In the last few years, while the mechanisms of bone derangement in CD have been extensively studied, less attention has been paid to the clinical management of this complication: there is in fact very little information available on the timing of the first bone mineral density (BMD) measurement, on follow-up frequency, even on the best treatment options.

## 2. Bone Damage and Mineral Metabolism Derangement in Celiac Disease

Intestinal malabsorption and inflammation contribute to the pathophysiology of bone damage in CD. Villous atrophy is responsible for alterations of intestinal absorption, and a negative calcium balance was shown in CD patients due to several mechanisms: malabsorption of calcium in untreated patients [16], partially reversible after gluten-free diet (GFD) [17]; the reduction of calcium intake [18] also due to a secondary lactose intolerance [19]; and the reduction of intestinal calcium absorption due to its binding to intraluminal unabsorbed fatty acids [16]. Hypocalcemia can induce a compensatory increase of serum levels of parathyroid hormone (PTH), in turn responsible for an increase of bone turnover [7,20]: in untreated CD, serum PTH correlates with markers of both bone synthesis, such as osteocalcin, as well as resorption, like telopeptide of type I collagen (ICTP) [5]. Bone resorption is faster than bone neoformation, resulting in net bone loss and a high turnover osteoporosis [21]. The increase of serum PTH enhances the activity of the renal enzyme 1-α-hydroxylase, which converts 25-vitamin D into 1,25 vitamin D, in order to improve calcium absorption at intestinal level. However, this effort is ineffective, mainly due to the lack in immature enterocytes of celiac mucosa of calbindin [22], a vitamin D-dependent calcium-binding protein, minimizing the role of vitamin D malabsorption [23]. Finally, high levels of 1,25 vitamin D might have the paradoxical effect of increasing bone resorption, as shown in patients with chronic renal failure [4]. Accordingly, vitamin D functions are rarely impaired in untreated CD, as hyperconversion of metabolite 25-vitamin D guarantees adequate levels of the active form 1,25 vitamin D.

Intestinal malabsorption could also lead to some deficits of other minerals, fat and water soluble vitamins that could affect normal bone metabolism. In particular, low levels of zinc were described in non-treated celiac patients [24], and related to low levels of insulin-like growth factor, that are subsequently responsible for derangement in bone metabolism, growth and immune function [25]. 

This complex network of events is present in both overt symptomatic and subclinical CD to be a disease that is below the threshold of clinical detection without signs or symptoms sufficient to trigger CD testing in routine practice—or it is silent, equivalent to asymptomatic CD patients, even if the extent of bone loss and the alterations of serum levels of indices of bone and mineral metabolism may be less severe than in CD patients with overt malabsorption [3]. 

More recently, much evidence has also suggested the role of both local and systemic inflammation in the pathophysiology of bone loss in CD, characterized by a chronic increase of both mucosal and serum pro-inflammatory cytokines, in particular TNFα, IL-1 and IL-6 [26,27]. IL-1 and TNFα stimulate osteoclastogenesis and bone resorption [28]; IL-6 has a pivotal role in bone resorption by recruiting osteoclast precursors and stimulating their differentiation [29]. In untreated CD patients, serum IL-6 levels inversely correlate with BMD [27] and directly with PTH and ICTP levels, a marker of bone resorption [30]. Recently, the existence of a complex cytokine imbalance in CD patients, affecting both osteoclast and osteoblast activity was shown: cultures of peripheral blood mononuclear cells of healthy donors with sera of untreated CD patients result in an increase in osteoclast number and IL-6 levels, together with an inhibition of IL-12 and IL-18 [31], two cytokines showing an *in vitro* inhibitory effect on osteoclastogenesis and osteoclast activity [32,33].

In the last 15 years, great attention has been given to the RANKL/RANK/osteoprotegerin pathway, that is today considered the main signaling system in bone metabolism. The receptor activator of nuclear factor κB ligand (RANKL) is expressed and secreted by osteoblasts; it binds RANK, located on the surface of osteoclast precursors, to induce the differentiation of these cells into mature osteoclasts, promoting bone resorption. Osteoprotegerin (OPG) is also secreted by osteoblasts; it acts as a decoy receptor for RANK and blocks RANK–RANKL interaction [34]. In CD patients, an increased level of OPG and RANKL was described, with an OPG/RANKL ratio significantly lower than controls. Moreover, the OPG/RANKL ratio was correlated with spine BMD [35] and with IL-6 levels [31]. 

The pathophysiological role of autoantibodies against OPG is also debated, as in a recent paper the presence of these antibodies was detected in a man with CD, high bone turnover and severe osteoporosis not responsive to GFD and to calcium and vitamin D supplementation [36]. This observation was not confirmed by a subsequent study on a large cohort of CD patients on GFD [37].

Further factors are linked to endocrine and reproductive disorders, commonly part of CD clinical presentation. In particular, early menopause and periods of amenorrhea could occur in women, due partly to malnutrition and partly to hormonal imbalance, and could worsen the severity of osteoporosis [38]. In men, hypogonadism was described, due to a reversible androgen resistance [39] and to hyperprolactinemia [40], and considered a possible adjunctive factor risk for osteoporosis [41]. Finally, CD is frequently associated with autoimmune thyroiditis and type I diabetes mellitus [42]: both these disorders are at high risk for osteoporosis [43,44].

### 2.1. Effect of GFD

Strict adherence to GFD allows BMD improvement but it is not able to normalize it in all cases. Mucosal recovery does not appear to be the only determining variable: in fact, with the same histological response, bone mass normalization is present in celiacs on GFD since early infancy [45] but not always in patients on GFD for the same length of time but diagnosed at a later age. In particular, normalization of BMD levels in childhood CD may be complete as early as after two years of GFD [46]. On the contrary, in adults, many cross-sectional studies demonstrated higher BMD levels in treated *vs.* untreated CD patients but still lower than in healthy volunteers [3,4,5,6,7,8,9,47,48,49,50]; also the prevalence of alterations of indices of bone and mineral metabolism is lower in treated *vs.* untreated patients [47,51,52]. These results were shown in patients on GFD for a median duration of 28.5 months [5], in patients on GFD from a mean of 3.6 years [47] and in a group of patients treated for a mean of 16 years, a very long period of GFD [52]. It is therefore evident that the early onset of bone damage, probably before achieving bone mass peak, is an important time point determining GFD-induced bone mass gain. Even longitudinal studies are of little help here, as the longest period of GFD evaluated was 5 years [53]. On the other hand, these studies have provided important information on the kinetic of bone mass recovery with the start of GFD and correlations with the modifications of bone-mineral metabolism parameters. Following a GFD with optimal compliance for a period of one year allows a significant improvement of BMD values, ranging from 5% [6] to 8% [3] according to different studies. These results were confirmed in a larger cohort of patients enrolled at diagnosis and restudied after one year of GFD [8]. In a two-year study, GFD improved not only bone mass but also serum levels of indices of bone and mineral metabolism. BMD improvement was more evident after two years than after one year of GFD, suggesting that a period longer than one year was necessary to point out intrinsic capacities of an individual patient to recover bone mass. Serum levels of propeptide of type I procollagen (PICP) at diagnosis proved to be a strong predictor of bone mass gain after two years, suggesting the possibility of selecting the group of patients with high levels of bone matrix formation activity that is more likely to readily respond to GFD [54]. In a three-year study, BMD increased in 92% of CD patients in GFD with a mean bone mass gain around 3%–4% per year. However, only 12% of patients showed a normalization of BMD. In particular, in a small group of patients, it was evident that relatively good bone mass gain was present during the first year, but was negligible in the subsequent study period [55]. These observations agree with a five-year study showing femoral and lumbar BMD values at five years similar to BMD values at one-year follow-up both in men and women, with the exception of trochanter values, which proved to be higher at five-year measurement than one-year values [53].

In summary, BMD values normalize only in children, when diagnosed early in infancy and if they follow a long-term GFD with optimal adherence. On the contrary, BMD values in adults show a good improvement in the first period, generally around two years, after the institution of a GFD; the improvement is then generally unsatisfactory and treatment with a mineral-active drug should probably be considered. Nevertheless, CD patients show a wide range of response to GFD and risk factors for osteoporosis include old age at diagnosis and the degree of osteopenia in late diagnosis, compliance to GFD, menstrual status, *i.e.*, late age at menarche, early menopause, periods of amenorrhea, low body mass index (BMI), low dietary calcium intake, inadequate physical activity and use of glucocorticoids [4,52,56,57]. What appears to emerge is that as age progresses and, in women as menopause approaches, the ability to recover bone mass seems to diminish, being greatest in childhood and lowest in peri- and postmenopausal women. In this latter subgroup of patients, waiting two or three years to determine the extent of GFD-induced bone mass gain could thus be incorrect and the start of treatment with a mineral-active drug should be earlier, probably at diagnosis. 

The availability of predictive markers of GFD-induced bone mass gain could be a solution for this problem, but the mechanism responsible for the unsatisfactory improvement is not completely clear. A persistent reduction of fractional calcium absorption was shown in patients on GFD, besides the improvement of intestinal mucosa architecture [17], and in a subgroup of patients the persistence of a secondary hyperparathyroidism and a significant correlation between serum PTH levels and femoral BMD were shown [17]. The possible role of secondary hyperparathyroidism was suggested by other papers [20,58] but also disproved [59], and the proposed pathophysiological mechanisms for the persistent raise of serum PTH were residual villous atrophy leading to calcium malabsorption [20], a reduction of calcium intake [18], but also a slow reversal of parathyroid hyperplasia [60]. Partial adherence to GFD [55] and incomplete mucosal recovery [57] could also have a role in subgroups of treated patients. 

Circulating factors secondary to persistent activation of the mucosal immune system could directly interfere with osteoclastogenesis and osteoblast activity. It was shown that in patients following GFD for a mean period of 40 months [31] the prevalence of bone damage is around 40%, and circulating levels of cytokines (IL-6, IL-1beta, TNF-alfa, TNF-beta, IL-12, IL-18, RANK-L, OPG) are significantly lower than in untreated patients, but significantly higher than in healthy volunteers. In particular, the osteoclastogenic activity of sera from patients on long-term GFD proved to be still significantly higher than sera of healthy volunteers and serum cytokine levels were not correlated to PTH levels [31]. An altered ratio between RANKL and OPG in untreated patients normalizes to healthy volunteer levels in patients on GFD [31,35]. Accordingly, the dietary treatment with GFD alone is not able to completely control the increased osteoclast differentiation and activity present in CD, as confirmed by a strong correlation between OPG/RANKL ratio and BMD [35], and the mechanism responsible for bone damage does not involve PTH. Finally, while a three-year period of GFD determines a significant decrease of IL 6, which is significantly inversely correlated at diagnosis with lumbar BMD, it cannot normalize IL-1β and IL-1 receptor antagonist serum levels [27]. 

Therefore, persistent inflammation in treated CD patients could have a role in the persistence of bone mass derangement. In particular, the predominant mechanism responsible for bone derangement seems different between short-term and long-term treated CD patients: in the period immediately after diagnosis, the malabsorption of calcium and the consequent hormonal and vitamin D alterations appear to be the prevalent pathophysiological mechanism, their correction allowing a satisfactory bone mass gain, comparable to the effect of administering mineral-active drugs in postmenopausal osteoporosis [61]. Unfortunately, the extent of the loss of bone mass in untreated CD is very often higher than the extent of the recovery induced just by GFD in the early stages of treatment and, once the GFD-induced metabolic surge that occurs in this phase is over, persistent bone loss seems due to the persistent activation of a local mechanism, related to chronic inflammation. Preliminary data from our group confirm this hypothesis, as an in-depth evaluation of hormonal and local factors suggests that high levels of OPG and low levels of PICP select the subgroup of CD patients with a persistent reduction of bone mass, despite strict adherence to GFD and architectural villi reconstitution [62]. If confirmed, these markers might be used to identify those patients who need mineral-active treatment associated with gluten-free diet.

## 3. Fracture Risk

BMD is only one of the factors that contribute to establishing the extent of fracture risk in osteoporotic patients. Other factors are related to bone mechanical characteristics, such as stiffness of cortical bone, but also to inadequacy of protectors from trauma (body mass, fat and muscle compartments) and to neuromuscular dysfunction [63]. 

Several studies pointed out the prevalence of fracture in celiac population, but with a very important heterogeneity in methods (study design, sample selection, fracture data collection) and cohorts studied (treated/untreated CD), making available data often inconsistent and difficult to interpret [64]. Most authors agree on the increased prevalence of fracture in CD patients [63,65,66,67,68,69,70,71] and a recent meta-analysis evaluating a total of 20,955 CD patients and 96,777 controls described a risk of fracture 43% greater in CD [72]. Data on fractures were collected by mailed questionnaires, by personal interviews or by medical records; consequently, results on peripheral fractures might be more easily estimated and axial fractures underestimated. Only a cross-sectional study explored the existence of asymptomatic vertebral fractures by spinal X-ray and did not find an increase of vertebral fractures in CD patients [68]. However, if CD patients are subdivided according to the clinical presentation, peripheral fracture risk proves to be higher than controls in patients with overt malabsorption symptom, while it is similar to the general population in subclinical and silent presentation [71]. These data were confirmed in a more recent case-control study, pointing out a higher peripheral fracture risk also in men and underlining again the importance of adherence to GFD [63]. However, to confirm what was said above with respect to pathophysiology, in a population-based study in Olmsted County, CD patients showed a fracture risk twice that of controls, and this figure persisted unchanged during GFD [66].

In general, however, large population-based studies should be interpreted with care, since, for example, in one study on the fracture risk in CD patients on a cohort of 1021 celiac patients, a possible misclassification of patients could have accounted for the negativity of results, as data were extracted from the National Patient Discharge Register, known for a low estimated validity of diagnosis of CD (78%) [73].

It is, finally, likely that, in addition to just BMD measurement, assessment of the physical characteristics of bone, such as its elasticity, can add something to our understanding of the mechanisms that favor fractures [74,75]. Unfortunately, no studies are available that correlate bone ultrasound densitometry parameters with fracture risk in CD patients.

## 4. Clinical Management

Only a limited number of international recommendations are available on the clinical management of osteoporosis in CD, probably due to the lack of sufficient data on patient follow-up and the role of the menopause. As already stated, there is no clinical or biochemical marker to select the subgroup of patients not responding to GFD alone with an improvement of bone mass and which will be characterized by a high risk of fractures; accordingly, we are not able, as yet, to optimize both treatment and timing of BMD follow-up measurement. 

In 2000, the British Society of Gastroenterology published the guidelines for osteoporosis in CD [76]. General advice aimed at modifying lifestyle factor risk was provided, such as enhancing physical activity, stopping smoking, avoiding alcohol excess. Moreover, a daily calcium intake of 1500 mg, even by pharmacological supplementation, and vitamin D supplementation, if inadequate serum levels were evident, was suggested. Bone densitometry was recommended at diagnosis for all patients, to detect osteoporosis early and to obtain the greatest possible benefit from treatment, or at least at menopausal age for women and at the age of 55 years for men. Postmenopausal women with normal BMD should repeat densitometry after two years. Osteoporotic postmenopausal women and men over 55 years should be offered treatment and bone densitometry yearly to monitor treatment efficacy. 

The subsequent awareness of an ultimately low absolute risk of fracture, even if higher than in the general population [77], determined strong criticism of the extensive use of bone densitometry at diagnosis, suggesting that BMD measurement should be restricted to patients with high short-term fracture risk, such as patients non-compliant with GFD or who failed to respond to dietary treatment, on glucocorticoid therapy, with untreated hypogonadism, older age, low BMI, and previous fragility fracture [78]. This led to reconsideration of the guidelines and the proposal of BMD measurement only in clinically non-responder patients, especially those with low BMI, in menopausal women and after 55 years for men [79]. In 2003, the American Gastroenterological Association guidelines on osteoporosis in gastrointestinal disease suggested that bone densitometry should be performed in adults with newly diagnosed celiac disease after one year of GFD, to allow for stabilization of bone density, implementation of GFD, calcium and vitamin D supplementation as needed, and, if necessary, bisphosphonates and hormonal therapy were strongly encouraged in osteoporotic patients [80]. 

A Canadian Position Statement on evaluation and management of skeletal health in CD was recently published [81]. BMD measurement was suggested at diagnosis only in adults with classic CD, and after one year of GFD in adults with asymptomatic or silent CD. The latter group of patients should be considered for earlier BMD evaluation in the presence of risk factors such as menopause, older age, history of fragility fracture, unexplained iron deficiency anemia, vitamin D deficiency/insufficiency, and high titers for CD serological markers. Indications for follow-up were also given: BMD should be re-evaluated after one year of GFD in the presence of osteopenia/osteoporosis at diagnosis, and after two years in cases of documentation of normal bone mass. The assessment of bone and mineral metabolism by dosing serum calcium, albumin, 1,25 dihydoxicolecalciferol and PTH levels should be repeated every six months until normalization. 

Clinical application of the Canadian guidelines does not, however, seem to allow substantial resource savings, and is thus very similar to the earlier British proposal. In patients with asymptomatic or silent CD with the aforementioned risk factors, early prescription of bone densitometry is indicated, since anemia and vitamin D alterations show a very high prevalence also in this subgroup. Considering the prevalence of bone loss in patients with and without overt malabsorption symptoms, BMD measurement could provide more important information in asymptomatic than in clinically overt malabsorber patients: bone loss is highly prevalent in overt malabsorption, and these patients could undergo mineral-active therapy as of diagnosis; conversely, asymptomatic/silent patients should be screened for bone loss presence. Moreover, the most important risk factor at diagnosis seems to be the age of the patient, and patients well over the age of peak bone mass could be treated without measuring BMD, while those patients below or shortly after the age of the peak should undergo BMD measurement. This approach seems the most correct one in optimizing the use of bone densitometry, but the problem remains for the free dispensing of the mineral-active drug, which in Italy depends on the presence of a pathological fracture. Optimization of this phase could be achieved by performing a radiological study of the lumbar spine in patients most at risk, such as symptomatic subjects, peri- and postmenopausal women and men over the age of 55, in order to detect vertebral fractures, together with a complete case history for previous fractures. In Italy (Lombardia Region), the cost of lumbar and femoral densitometry is €88.66 (€44.33 for each segment), while the cost of a lumbar spine X-ray is only €34.80. 

**Figure 1:**
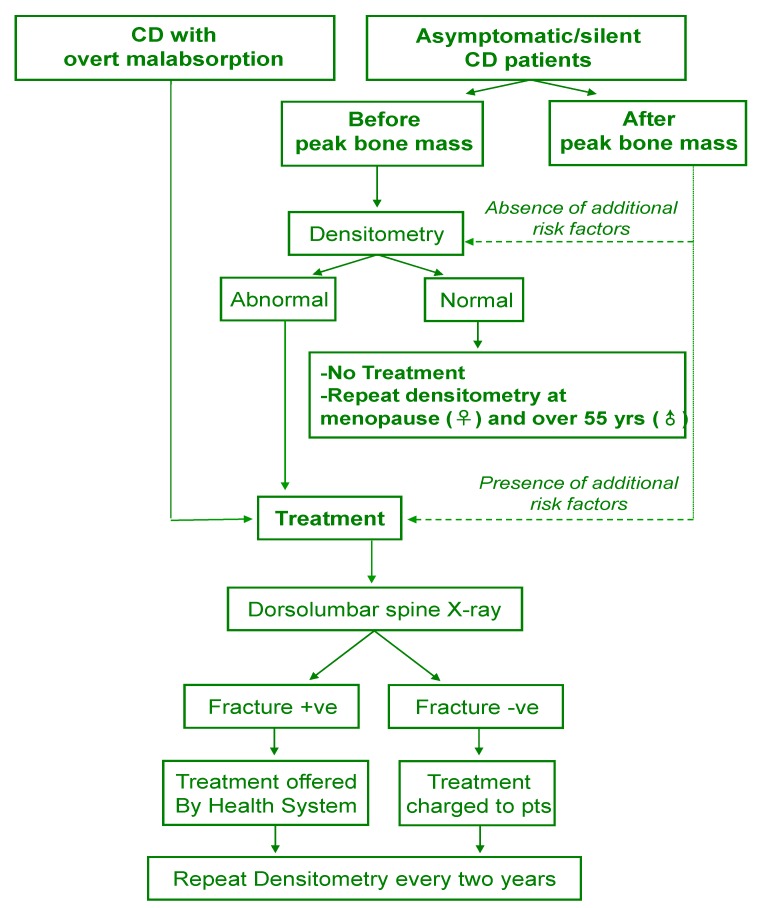
Diagnostic and therapeutic approach to CD patients without a previous fracture.

In conclusion, there is no general agreement on the correct timing of bone densitometry in celiac patients; screening at diagnosis seems to be not justified in all patients and the proposed alternative approach is explained in Figure 1. 

Treatment with a mineral-active drug in association to GFD, even without BMD measurement, should be prescribed to patients at high short-term risk of fracture, including symptomatic patients, asymptomatic patients in peri and postmenopausal period, men older than 55 years, low calcium intake, low BMI, poor compliance to GFD, or unresponsiveness to GFD following steroid therapy; in these cases, measure BMD after two years of treatment. To allow the free dispensing of the drug, patients should be screened for the presence of fracture and X-ray of the spine could be adequate to this aim. Young asymptomatic patients with a normal BMD should be re-evaluated at peri-menopausal period (female patients) or over 55 years (male patients).

As regards the choice of the drug for the treatment of osteoporosis in CD, there are no longitudinal studies dealing with this topic and we have no information on which to base our choice in the individual subgroups of patients. The current approach is clearly based on post-menopausal osteoporosis treatment with a choice between a weekly administration of alendronate and a monthly administration of ibandronate. The use of denosumab, a decoy receptor for RANKL able to reduce the activation of the osteoclast system, seems very interesting, also on the basis of the data obtained on the RANK/RANKL/OPG system. The results of the first studies are awaited in order to define the best strategy for the different types of CD patients.

## 5. Conclusions

In conclusion, in the pathophysiology of bone derangement in CD patients, both malabsorption and the persistent activation of inflammation at intestinal level are important, in a two-step model. Biomarkers with a predictive role of the normalization of BMD levels are needed and the evaluation of the RANK/RANKL/OPG system could offer some inputs on this topic.
